# Adoptive immunotherapy with engineered T cells expressing and HLA-A2 restricted affinity-enhanced TCR for LAGE-1 and NY-ESO-1 in patients with multiple myeloma following auto-SCT

**DOI:** 10.1186/2051-1426-1-S1-P30

**Published:** 2013-11-07

**Authors:** Aaron P Rapoport, Edward A Stadtmauer, Dan T Vogl, Brendan Weiss, Gwendolyn K Binder-Scholl, Dominic P Smethurst, Jeffrey Finklestein, Irina Kulikovskaya, Minnal Gupta, Joanna E Brewer, Alan D Bennett, Andrew B Gerry, Nick J Pumphrey, Helen K Tayton-Martin, Lilliam Ribeiro, Ashraf Z Badros, Saul Yanovich, Gorgun Akpek, Naseem Kerr, Sunita Philip, Sandra Westphal, Levine L Bruce, Bent K Jakobsen, Carl H June, Michael Kalos

**Affiliations:** 1 The Greenebaum Cancer Center, University of Maryland, Baltimore, MD, USA; 2 Abramson Cancer Center, Perelman School of Medicine, University of Pennsylvania, Philadelphia, PA, USA; 3 Adaptimmune Ltd, Oxford, UK; 4 Department of Pathology, Perelman School of Medicine, University of Pennsylvania, Philadelphia, PA, USA; 5 Translational and Correlative Sciences Laboratory, Perelman School of Medicine, University of Pennsylvania, Philadelphia, PA, USA

## 

We report on a 26 patient phase I/II clinical trial (NCT01352286) to investigate the safety, feasibility and anti-tumor activity of T cells engineered to express an affinity-enhanced TCR that recognizes the NY-ESO-1/LAGE-1 peptide complex HLA-A*0201-SLLMWITQC. Patients with high risk or relapsed, NYESO-1 and/or LAGE-1 positive multiple myeloma (MM), are infused with 1-10x10^10^ autologous TCR-engineered T cells 2 days after autologous stem cell transplantation (aSCT). CD25 depleted CD4/CD8 T cells are activated and expanded by anti-CD3/28 beads, and modified by lentivector. Disease response is evaluated in accordance with IMWG criteria. Blood and marrow is monitored for the persistence and phenotype of engineered cells; marrow is monitored for expression of target antigen. 20 patients (average age of 57) were infused with an average of 8.7 x 10^9^ T cells (range 4.5 x 10^8^ - 4.2 x 10^9^), transduced at an average of 34% (range 18% - 49%). Patients received a median of 2 prior therapies including 6 with prior transplant. 50% of tumors contained high risk chromosomal abnormalities, and NY-ESO expression is correlated with adverse prognosis. Patients with disease just prior to transplant (14/17) were assessed for response by day 100 (Table 1). Infusions were well tolerated with no cardiac-related toxicity. At least possibly related grade 3/4 events included blood and marrow or GI toxicity, all of which resolved. Engineered T cells expanded and persisted in blood and marrow at 180 days by Q-PCR and flow-cytometry in all but one case. 7 patients progressed after day 100, which was accompanied by loss of engineered T cells or loss of tumor antigen. The response by day 100 compares favorably to historic responses in patients undergoing first or second transplant, which is encouraging in this advanced stage population. We are investigating correlates of response and mechanisms of relapse.

**Table 1:** 

Best Response – D100	# Pt	%
CR	1	7% (1/14)
nCR	10	72% (10/14)
VGPR	0	0
PR	3	21% (3/14)
SD	0	0
PD	0	0
Total	14	100%

